# Occlusion of faces by sanitary masks improves facial attractiveness of other races

**DOI:** 10.3389/fpsyg.2022.953389

**Published:** 2023-01-12

**Authors:** Miki Kamatani, Yuki Miyazaki, Jun I. Kawahara

**Affiliations:** ^1^Faculty of Humanities and Human Sciences, Hokkaido University, Sapporo, Japan; ^2^Department of Psychology, Fukuyama University, Hiroshima, Japan

**Keywords:** sanitary mask, COVID-19, own-race, other-race, attractiveness, experience

## Abstract

Recent studies provide mixed results regarding whether the perception of facial attractiveness is increased or decreased by partial occlusion with a sanitary mask. One set of studies demonstrated that occluding the bottom half of a face increased facial attractiveness. This effect is thought to occur because the occluded area is interpolated by an average facial representation that is perceived as attractive. However, several groups of studies showed that partial occlusion can increase or decrease perceived attractiveness depending on the attractiveness of the original (unoccluded) face, due to regression to the mean. To reconcile this inconsistency, we propose that the occluded area is interpolated not by an average facial representation, but by a template of moderate attractiveness, shaped by the distribution of each viewer’s experience. This hypothesis predicts an interaction between occlusion and the attractiveness of the original face so that occluded attractive faces are rated as less attractive, while occluded unattractive faces are rated as more attractive. To examine this hypothesis, the present study used attractiveness-rating tasks with mask-free versus masked faces in own-race and other-races categories. Viewers were familiar with own-race faces and unfamiliar with other-races faces. If moderate-attractiveness interpolation were the explanatory factor, the interaction between the occlusion and the attractiveness of the original face should be found only in the rating of own-race faces. Consistent with this hypothesis, the interaction between the occlusion and the attractiveness of the original faces was significant only for the own-race faces. Specifically, wearing a sanitary mask decreased the facial attractiveness of attractive faces in the own-race, while it increased the attractiveness regardless of the level of facial attractiveness in other-races. These findings suggest that the occluded area of own-race faces is interpolated by a facial template of moderate attractiveness. The other-races template could be developed using familiar exemplars such as celebrities. Thus, interpolation by such a template should result in elevated attractiveness relative to that by an own-race template. Accordingly, the apparent inconsistency in the literature regarding the effect of partial occlusion on physical attractiveness can be explained in terms of differences in the template involving interpolation of the occluded area.

## 1. Introduction

Faces convey a large amount of information during human interactions. The COVID-19 pandemic resulted in an increase in the use of sanitary masks worldwide. Face masks offer protection against disease by blocking the inhalation and exhalation of respiratory virus particles (e.g., Asadi et al., 2020; Chu et al., 2020; Li et al., 2021). However, they are disadvantageous in terms of interpersonal cognition and communication. For example, sanitary masks impede identification of individuals (Carragher and Hancock, 2020; Freud et al., 2020; Noyes et al., 2021) and recognition of emotional expressions (Carbon, 2020; Grundmann et al., 2021; Noyes et al., 2021; Parada-Fernández et al., 2022), and also affect the perceived facial attractiveness of the masked individual (Miyazaki and Kawahara, 2016; Patel et al., 2020; Kamatani et al., 2021; Hies and Lewis, 2022). These disadvantages are attributable to occlusion of the lower half of the face by the mask.

A set of recent studies revealed a systematic effect of occlusion of faces by masks on the perception of facial attractiveness. Specifically, a recent study (Patel et al., 2020) revealed that masked faces were perceived to be more attractive than the original faces. Participants viewed both a mask-free face and a masked face and rated the attractiveness on a scale of 1 (least attractive) to 10 (most attractive). The results revealed that masked faces were perceived as more attractive compared to mask-free faces when the original faces were unattractive or average in terms of attractiveness. Hies and Lewis (2022) also reported improvements in scores for masked faces in comparison to those that were mask-free. A similar effect was found to demonstrate that occluded faces were generally rated as more attractive than unoccluded faces (Sadr and Krowicki, 2019; Orghian and Hidalgo, 2020). Orghian and Hidalgo (2020) suggest that attractiveness is enhanced by an interpolation process. Some studies suggest that the average face is not the most attractive (e.g., Alley and Cunningham, 1991; Etcoff et al., 2011). However, given that facial averageness reflects developmental stability, and heterozygosity provides advantages for mating, it is intuitive that average faces created by morphing multiple images are perceived as attractive (e.g., Langlois and Roggman, 1990; Grammer and Thornhill, 1994 Rhodes and Tremewan, 1996; Apicella et al., 2007). Thus, by interpolating the occluded area with a representation of an average face, the resulting composite should be perceived more attractive than the unoccluded original. However, recent findings showed that this was not always the case.

A different group of researchers found an occlusion effect, but it was affected by the attractiveness of the original faces (Miyazaki and Kawahara, 2016; Kamatani et al., 2021). Specifically, Kamatani et al. (2021) found that the wearing of a sanitary mask increased the facial attractiveness of masked unattractive faces in comparison to mask-free faces. However, the mask also decreased the attractiveness of attractive faces. This effect of wearing masks occurs because the occlusion reduces the cues contributing to facial attractiveness. For example, a mask can occlude features related to low attractiveness such as asymmetric facial contours, misaligned or distorted facial features (e.g., Rhodes et al., 1998, 1999; Scheib et al., 1999 Little and Jones, 2003), and pimples and scars (e.g., Jaeger et al., 2018). Thus, negative ratings may trend toward the mean value. On the contrary, a mask on an attractive face can occlude features related to high attractiveness such as symmetric facial contours and smooth skin; thus, positive ratings may also trend toward the mean value.

Although the effect of occlusion by sanitary masks on perceived facial attractiveness, as reviewed above, has been reported (Miyazaki and Kawahara, 2016; Patel et al., 2020; Kamatani et al., 2021; Hies and Lewis, 2022), the occlusion effects of sanitary masks were inconsistent across studies. Importantly, Patel et al. (2020) and Hies and Lewis (2022) demonstrated a general improvement in attractiveness ratings by occlusion regardless of the attractiveness of the unmasked faces, whereas Kamatani et al. (2021) and Miyazaki and Kawahara (2016) found an interaction in that occlusion decreased the attractiveness of attractive faces and increased that of unattractive faces. This interaction cannot be explained by the same interpolation of an average face explaining why a general improvement in facial attractiveness is observed with wearing a mask. Likewise, the general improvement in attractiveness cannot be explained by a reduction of perception of cues of facial attractiveness.

We argue that the mixed results are attributable to the occluded area of a masked face being interpolated not by the representation of an average face, perceived as attractive, but rather by the representation of a face with moderate attractiveness. The findings of Miyazaki and Kawahara (2016) and Kamatani et al. (2021) support this idea. In their study, the participants and individuals presented in the stimuli were Japanese. The participants would develop a fine-tuned facial template representing a Japanese face of moderate attractiveness (Zhou et al., 2016). Instead of developing a single template of an averaged image, accumulated statistical evidence indicated that the participants saw moderately attractive faces most frequently. They were shown an ample number of moderately attractive features, including those with symmetric contours, typical global arrangements of facial parts, and smooth surface textures. In other words, viewing multiple faces rated as moderately attractive yielded statistical scores reflecting moderate attractiveness (scores of 40–50 on a scale of 1–100), rather than high attractiveness (scores of 90–100). This does not contradict previous research indicating that averaged faces are perceived as attractive. Instead, viewing multiple faces rated 40–50 on the attractiveness scale, i.e., viewing moderately attractive faces more frequently, results in the viewer imagining moderately attractive (ratings of 40–50) faces (or facial features) when asked to imagine or interpolate a face. This is distinct from the idea that viewing multiple faces leads to a single merged image. The participants could create such a template through observing faces of people in own-race group (Zhou et al., 2016). Therefore, when they encounter a masked face, they might have interpolated the occluded half using their moderate-attractiveness template. The use of a moderate-attractiveness template is reasonable in this case because it would represent the most statistically probable samples and has been shown to reduce modification error costs between predictions and real faces when the faces were revealed. However, this would not be likely to be the case when participants in previous studies were assumed to be recruited from diverse populations (Patel et al., 2020) and the stimuli contained racially heterogeneous faces (Patel et al., 2020; Hies and Lewis, 2022). In those cases, the participants would be expected to create a moderate-attractiveness template based on their experience of individuals with the same and different racial backgrounds. They would be likely to accumulate fine-tuned facial representations of individuals with the same racial background, but less likely to create a moderate-attractiveness template of the same quality for those of a different race, because they would be less sensitive to the fine details of faces of people of different races (Cross-Race effect; Apicella et al., 2007; Hourihan et al., 2012). Instead, they might have aggregated available knowledge from memory or been biased by other-race representatives such as celebrities (e.g., movie stars, athletes, and politicians). Consequently, the resulting viewer’s moderate-attractiveness template could be biased toward attractive faces. Therefore, when the participants encountered a masked face, they may have interpolated the occluded half using a template biased toward attractive faces, resulting in an increase in the perception of attractiveness.

**Figure 1:**
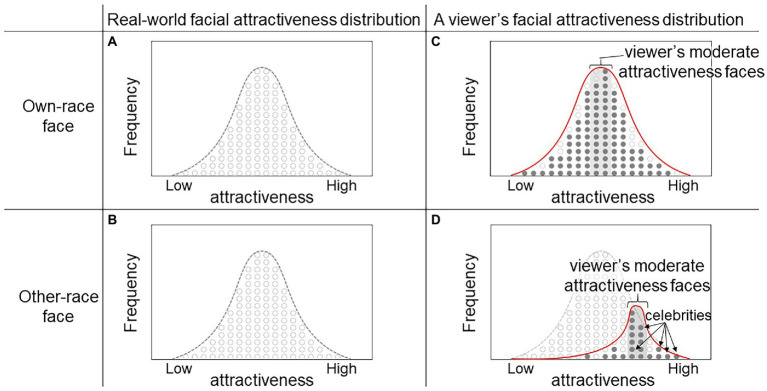
Distribution of real-world own-race **(A)** and other-race **(B)** facial attractiveness ratings. **(C)** Distribution (red solid line) of facial attractiveness ratings based on the viewer’s own experience. The ordinate represents the frequency and the abscissa represents facial attractiveness ratings. Filled circles represent encountered individuals. Own-race moderately attractive faces are viewed most frequently. **(D)** The distribution (red solid line) of other-race facial attractiveness ratings is skewed toward high attractiveness because the viewer’s experience is limited to attractive faces, such as celebrities. A viewer sees other-race highly attractive faces more often than moderately attractive faces.

This idea is depicted in Figure 1. In this figure, the ordinate is frequency and the abscissa is facial attractiveness. The left two panels represent distributions of facial attractiveness of own-race (Figure 1A) and other-race (Figure 1B) individuals in the real world. Specifically (Figure 1A) represents the “real-world facial attractiveness” as a hypothetical dataset containing accumulated measurements of attractiveness scores rated by people in a given race group and (Figure 1B) represents “real-world facial attractiveness” as rated by people in a different race group. In Figure 1C shows a viewer’s internal representation of the facial-attractiveness distribution of facial attractiveness generated *via* their own experience so that they see moderate attractive faces most frequently. The filled circles represent the encountered individuals. In Figure 1C, a viewer sees own-race faces of moderate attractiveness most frequently and encounters extremely attractive faces very rarely (extremely unattractive faces, as well). According to the Law of Large Numbers, the range of moderately attractive faces that the viewer conceives approximates to the range of moderate attractiveness in the real-world population. However, this does not apply to the faces of people of other races (Figure 1D). Viewers in environments with limited opportunities to see other-race faces would encounter a biased sample of attractive faces, such as movie stars, athletes, and politicians who are rated as highly attractive. This is because the number of samples of other-race faces that the viewer encounters is very small and biased toward attractive faces. The resulting distribution is skewed toward the highly attractive direction (right end of abscissa in Figure 1D), increasing the level of moderate attractiveness that functions as the viewer’s standard.^1^

To examine this interpolation as explained by the moderate interpolation hypothesis, that is, the attractiveness rating of a masked face depends on its grouping (own-race or other-races), we established an experimental design in the present study that compares the attractiveness ratings of mask-free vs. masked faces between an own-race (Experiment 1: Japanese participants rated Japanese female faces) and other-races (Experiments 2–4: Japanese participants rated Black, White, and non-Japanese Asian female faces, in separate experiments). We focused on how the face template used for interpolation differs based on race. Sanitary masks affected attractiveness ratings for male faces similarly to female faces (Miyazaki and Kawahara, 2016; Hies and Lewis, 2022; Pazhoohi and Kingstone, 2022, Experiment 1). Therefore, we considered that only female faces used as the face stimuli were sufficient to examine the hypothesis of the present study. If the participants interpolated occluded faces based on a moderate-attractiveness template, masked other-races faces should be perceived as more attractive regardless of the baseline attractiveness because the moderate-attractiveness template of other-races is biased toward high attractiveness. Therefore, we predicted that the masked faces of other-races should be perceived as more attractive than the mask-free faces of those races. On the contrary, if the moderate-attractiveness template is involved in the interpolation process for masked faces in the own-race, the rating scores should reflect an interaction with the baseline attractiveness (i.e., improvement for unattractive faces and impairment for attractive faces).

There are two unresolved issues. First, the present model, informed by a moderate interpolation hypothesis, is based on the frequency with which viewers encounter faces of other races. This issue was addressed using a survey designed to validate such encounters. Second, the interpretation may not be consistent. The results of attractiveness ratings of masked other-races faces being greater than mask-free faces cannot be interpreted as an improvement in attractiveness due to the wearing of masks on those faces. Instead, another possibility is that those mask-free faces might be rated less attractive (i.e., devalued) due to an increase in exclusive attitudes toward persons from abroad as the COVID-19 pandemic expands (Yamagata et al., 2020), probably because individuals without masks are seen as a threat to anti-infection policies. For example, Yamagata et al. (2020) found an association between infection-avoidance tendencies and strong exclusionary attitudes towards persons from abroad. Therefore, we included the perceived vulnerability to disease (PVD) scale to examine whether the attitude of infection avoidance was related to negative ratings for mask-free faces. The PVD scale includes 15 items grouped into two internally consistent subscales, perceived infectability and germ aversion. The perceived infectability subscale assesses beliefs related to one’s own susceptibility to infectious disease and the germ aversion subscale assesses beliefs related to one’s emotional discomfort toward a high potential for pathogen transmission (Duncan et al., 2009; Fukukawa et al., 2014). We predicted that concern about infectious diseases would be negatively related to the perceived attractiveness of mask-free other-races faces. Here, people who report higher PVD scores should perceive mask-free other-races faces as less attractive relative to others who report lower PVD scores.

## 2. Materials and methods

### 2.1. Participants

One hundred and fifty-six Japanese undergraduate and graduate students participated. They were assigned to the aforementioned four experiments (Experiment 1, *N* = 60, *M* = 18.37 years, *SD* = 0.63, 31 females; Experiment 2, *N* = 32, *M* = 20.50 years, *SD* = 2.29, 15 females; Experiment 3, *N* = 32, *M* = 18.50 years, *SD* = 0.82, 19 females; Experiment 4, *N* = 32, *M* = 18.78 years, *SD* = 1.65, 21 females). Participants in Experiment 2 were recruited from a participant pool at Hokkaido University. Experiments 1, 3 and 4 were implemented as coursework at the Health Sciences University of Hokkaido. Thus, the number of study participants depended on class attendance. The number of participants in Experiment 2 was equal to that in Experiments 3 and 4. All participants provided informed consent. No one participated in more than one experiment. The experimental protocol of the present study was approved by the ethical review board of Hokkaido University (31–2, 3–02). The experiments were conducted from June 26, 2020 until September 10, 2020, and on July 2, 2021.

### 2.2. Apparatus and stimuli

Stimuli were presented in a web browser of each participant’s personal computer in Experiments 1, 3, and 4, and laboratory computer in Experiment 2 and were controlled by lab.js software (Henninger et al., 2022). Therefore, the size of the presented stimuli differed across experiments and participants, although no participants completed the experiment using their smartphones and tablets.

**Figure 2:**
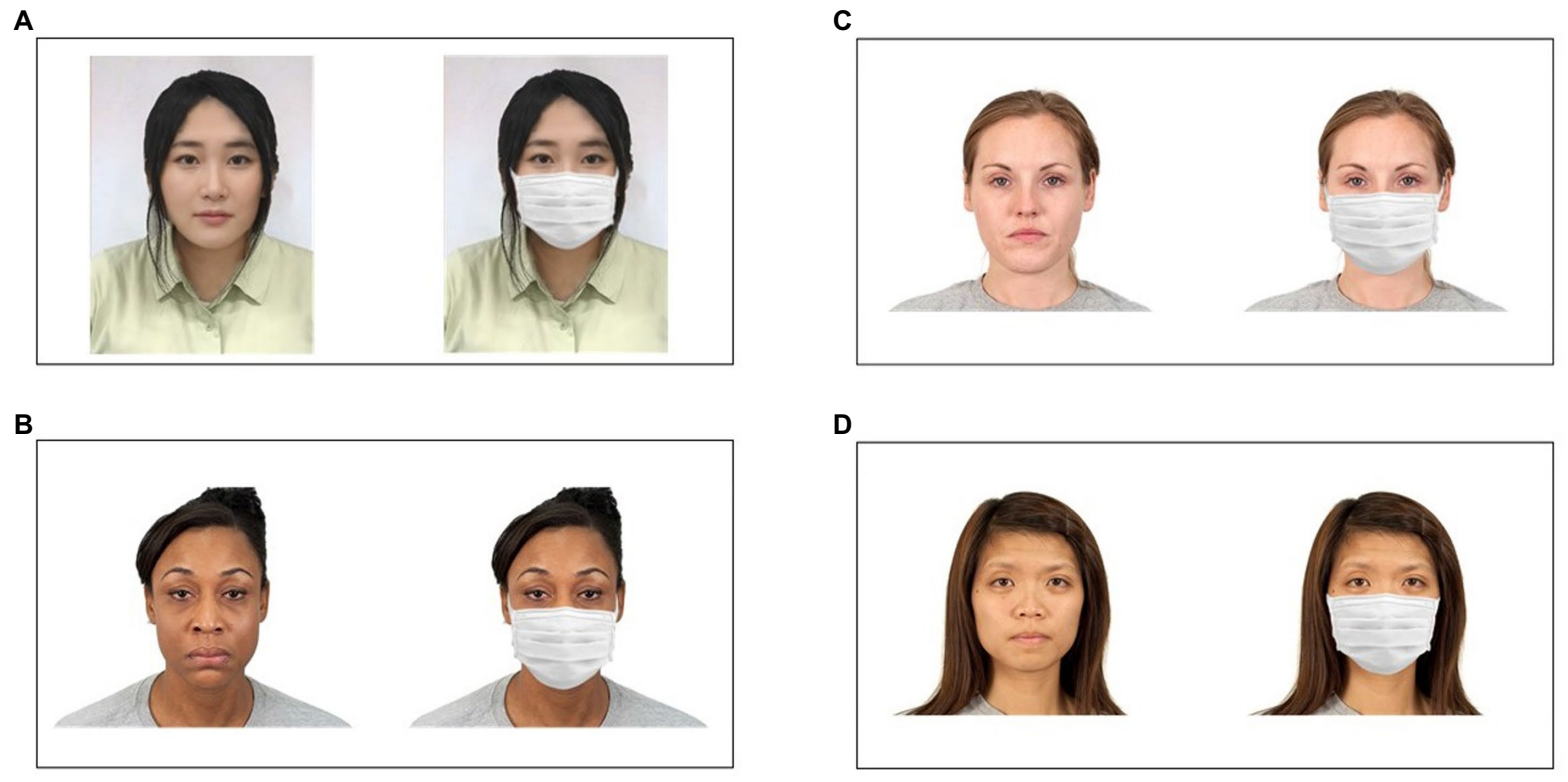
Examples of mask-free and masked faces. **(A)** Japanese females. **(B)** Black females. **(C)** White females. **(D)** Non-Japanese Asian females. The Japanese female faces (shown in panel **A**) were not used in the present study. Black, White, and non-Japanese Asian female faces shown in panels **B–D** were used in the present study.

Different sets of faces images were used across experiments. For Experiment 1, Japanese female facial images [354 × 472 pixels; 8.1 cm (W) × 10.8 cm (H) on a 14-in laptop screen, color JPEG format] were selected from a homemade database of young female Japanese facial images (Kawahara and Kitazaki, 2013). For Experiments 2–4, Black, White, and non-Japanese Asian facial images [2,444 × 1,718 pixels; 11.0 cm (W) × 7.7 cm (H) on a 14-in laptop screen, color JPEG format] were selected from the Chicago Face Database (Ma et al., 2015)^2^ based on attractiveness ratings obtained for 1,087 participants (516 Whites, 117 Asians, 74 Blacks, 72 bi- or multi-racial, 57 Latinos, 18 other, and 233 unreported races). Image size varied among the databases, so the image sizes in Experiment 1 were different from those in Experiments 2–4. A total of 48 images were selected from each image set so that 16 images were included in three attractiveness categories (low, middle, and high). All face images included the hair and had a neutral expression or slight smile. Both databases include slightly smiling faces, the frequency of which did not vary according to attractiveness. Therefore, it is unlikely that the attractiveness ratings were reduced as a result of shielding, which could have prevented positive facial expressions from being distinguished. Furthermore, a previous study showed the same results for both facial image databases with slightly smiling faces, and for a control database of faces with no expressions (Miyazaki and Kawahara, 2016, Studies 1a and 2). None of the faces wore eyeglasses. There were no lighting-related differences in facial shading between the two databases. The background was white for both databases; however, it was slightly darker for the Japanese dataset. The Chicago Face Database images were fully frontal, while some of the Japanese faces were at a slight angle. The procedure to create masked faces was identical to that in Miyazaki and Kawahara (2016). Namely, an image of a white sanitary mask was superimposed on the face. The superimposition and removal of unnatural edges between the face and mask was conducted using graphic editing software (Adobe Photoshop 2020). Figure 2 represents a sample image of masked and mask-free faces.

### 2.3. Procedure

In each experiment, a single race of facial images was presented: female Japanese faces for Experiment 1, female Black faces for Experiment 2, female White faces for Experiment 3, and female non-Japanese Asian faces for Experiment 4. In every trial, a single face was presented in the center of the screen, concurrently accompanied by a horizontal rating scale located below the image. Participants rated the attractiveness of the facial image from 1 (very unattractive) to 100 (very attractive) by moving the slider on the rating scale and clicked the submit button to report the score. The inter-trial interval was 500 ms.

Forty-eight facial images of different individuals were presented to each participant in random order. As shown in the Figure 2, half of the facial images were masked faces, and the others were mask-free faces. No participants viewed the same individual twice for both the mask-free and masked conditions. The presence or absence of masks was counterbalanced according to participant identity. The Japanese version of the PVD questionnaire (Fukukawa et al., 2014) was administered after the completion of the rating tasks of Experiments 2, 3, and 4. We did not administer the PVD questionnaire after Experiment 1 because we were not aiming to assess the relationship between the scale score and the rating score of attractiveness in the own-race condition. Moreover, our unpublished data found no correlations between PVD scores and attractiveness rating scores of masked and mask-free faces (Supplementary material A). Participants scored each item from 1 (strongly disagree) to 7 (strongly agree).

### 2.4. Statistical analysis

All analysis were conducted using R software (version 4.0.3.) and all Analysis of Variance (ANOVA) tests were performed using the anovakun function in R. In the analyses of correlations between PVD scores, included subscale scores, and the rating scores of mask-free and masked faces, five participants were excluded because they did not answer the PVD questionnaire. We computed the sum of scores for each participant.

## 3. Results

### 3.1. Attractiveness ratings

**Figure 3:**
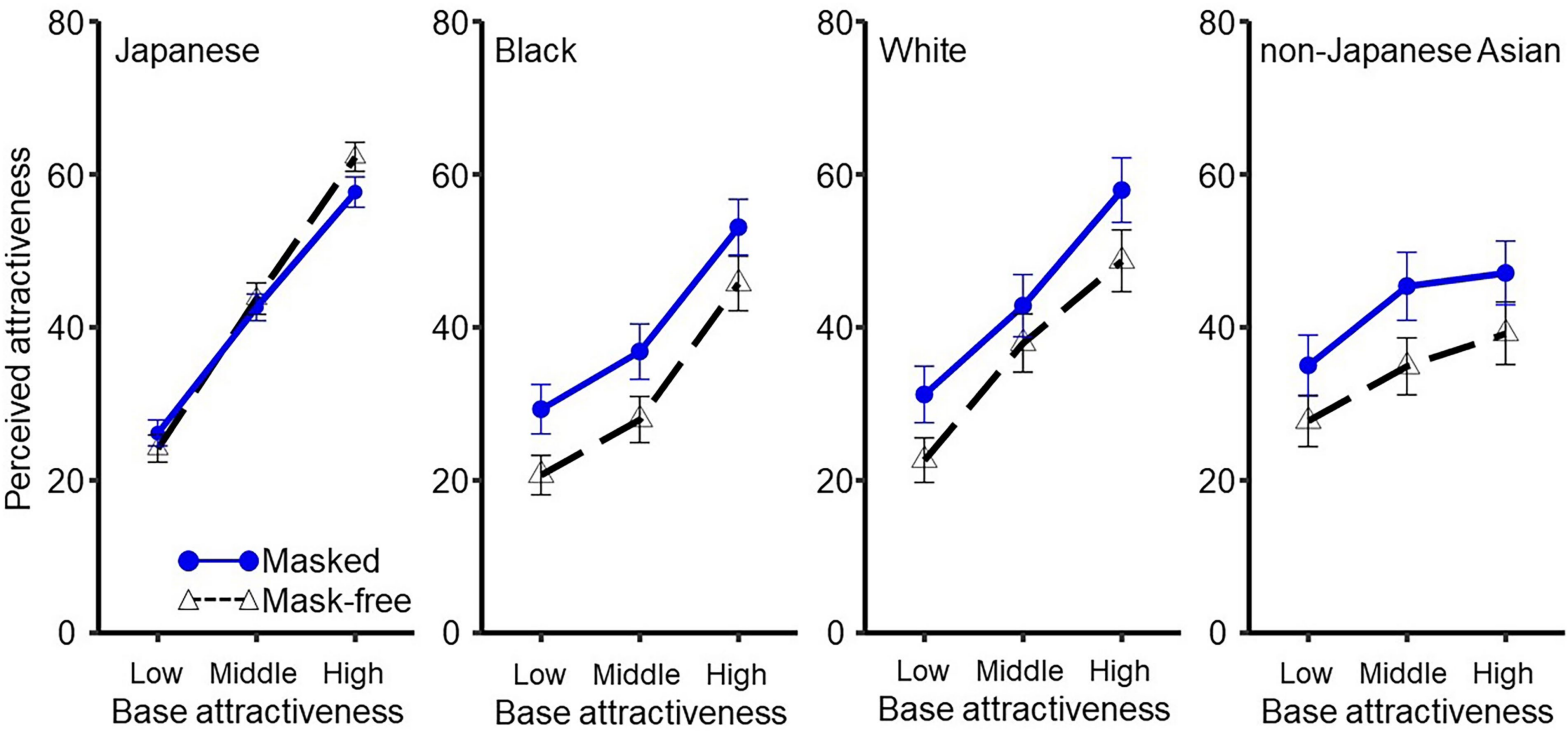
Attractiveness rating scores for masked and mask-free faces in each experiment. From left to right: Japanese, Black, White, non-Japanese Asian. Error bars represent standard errors.

The mean rating scores were plotted as a function of baseline attractiveness for masked (solid line) and mask-free (dashed line) faces in each panel, as shown in Figure 3. A two-way (baseline attractiveness × mask presence) ANOVA with a within-participant design for the rating scores of Japanese faces (i.e., own-race faces) revealed a significant main effect of baseline attractiveness [*F*(2,118) = 265.059, *p* < 0.001, η_p_^2^ = 0.817]. Holm’s test on this main effect revealed that the attractiveness rating was greater with an increase in baseline attractiveness [*t*s(59) > 13.824, *p*s < 0.001, *r*s > 0.875]. The main effect of mask presence was not significant [*F*(1,59) = 0.842, *p* = 0.362, η_p_^2^ = 0.014]. Importantly, the interaction between baseline attractiveness and mask presence was significant [*F*(2,118) = 8.691, *p* < 0.001, η_p_^2^ = 0.128]. Multiple comparisons of this interaction revealed that a sanitary mask decreases the facial attractiveness of faces with high attractiveness scores [*F*(1,59) = 7.648, *p* = 0.007, η_p_^2^ = 0.114]. For faces with low and middle attractiveness scores, the differences in perceived attractiveness between the masked and mask-free conditions were not significant [low: *F*(1,59) = 3.524, *p* = 0.065, η_p_^2^ = 0.056; middle: *F*(1,59) = 0.323, *p* = 0.571, η_p_^2^ = 0.005]. For Black, White, and non-Japanese Asian faces (i.e., other-races faces), the main effects of baseline attractiveness [*F*s(2,62) > 56.505, *p*s < 0.001, η_p_^2^s > 0.645] and mask presence [*F*s(1,31) > 17.131, *p*s < 0.001, η_p_^2^ > 0.355] were significant. Holm’s tests revealed that the attractiveness rating was greater with an increase in baseline attractiveness [*t*s(31) > 3.202, *p*s < 0.005, *r*s > 0.499]. For other-races faces the interaction between baseline attractiveness and mask presence was not significant [*F*s(2,62) < 2.042, *p*s > 0.138, η_p_^2^s < 0.061].

Overall, own-race facial attractiveness ratings decreased when a sanitary mask was worn. In contrast, facial attractiveness ratings for other races increased with sanitary masks, regardless of baseline facial attractiveness. The results for other races were identical to those obtained by rearranging the stimuli into three attractiveness categories for the 153 adults living in Japan (Supplementary material B). These results were consistent with our prediction based on the moderate interpolation hypothesis. Regarding the results of own-race facial attractiveness, the present study failed to demonstrate an exact replication of the findings of Kamatani et al. (2021), in that masked unattractive faces were perceived to be more attractive, whereas masked attractive faces were perceived to be less attractive. However, another study (Morioka, 2022) found comparable interactions consistent with the results of Kamatani et al. (2021). Nonetheless, it is notable that the decrement of attractiveness in highly attractive faces was more pronounced relative to the increment of attractiveness in low-attractive faces. This pattern in the results is consistent with our previous data (Kamatani et al., 2021). Therefore, this asymmetry would be a common feature involved in this interaction.

### 3.2. Frequency of encounters with other-race faces

We conducted a survey to determine the frequency with which viewers encounter faces of other races. A group of 30 newly recruited Japanese undergraduate and graduate students participated (*M* = 19.24 years, *SD* = 1.84, 17 females). The participants never experienced Experiments 1, 2, 3, and 4. Stimuli were presented on a 24-inch LCD monitor (100-Hz refresh rate, 1920 × 1,080 pixels) and controlled by a Linux PC using MATLAB software (The MathWorks) with the Psychophysics Toolbox (Kleiner et al., 2007).

Forty facial images were used as stimuli in the present experiment; 10 were randomly selected from each race. Black, White, and non-Japanese Asian face images were resized to match the Japanese face images, i.e., 354 (W) × 472 (H) pixels. First, 10 Japanese face images were presented sequentially in the center of the screen at a rate of 3,000 ms/image, with a blank display of 500 ms between stimuli. A single other-race face was presented in the center of the screen, accompanied by a horizontal rating scale located below the image. Participants indicated the frequency of encounters with the different races on a scale ranging from 1 (never) to 100 (as frequently as Japanese faces). We conducted a one-sample *t*-test to analyze the mean race-specific scores. The results showed that the participants encountered individuals of other races infrequently (Black: *M* = 26.253, *t*(29) = −30.206, *p* < 0.001, *r* = 0.985; White: *M* = 30.576, *t*(29) = −31.553, *p* < 0.001, *r* = 0.986; non-Japanese Asian: *M* = 51.073, *t*(29) = −15.391, *p* < 0.001, *r* = 0.944). Since new participants were recruited, it was not possible to confirm whether the participants in Experiments 1–4 had less contact with people from other races. However, the results supported the moderate interpolation hypothesis. In particular, Japanese individuals had opportunities to form a detailed template for own-race faces, but lacked this opportunity for other races because of the low encounter frequency.

### 3.3. Correlations between the rating score and perceived vulnerability to disease scores

Cronbach’s alpha for all 15 items was 0.74 (0.82: Duncan et al., 2009; 0.97: Fukukawa et al., 2014), Cronbach’s alpha for the perceived infectability subscale was 0.86 (0.87: Duncan et al., 2009; 0.87 Fukukawa et al., 2014), and Cronbach’s alpha for the germ aversion subscale was.75 (0.74: Duncan et al., 2009; 0.67 Fukukawa et al., 2014).

We calculated Pearson’s correlation coefficients between the PVD score and rating scores of masked and mask-free faces. There was a significant positive correlation between the PVD score and the rating score for masked Black faces (*r* = 0.365, *p* = 0.043), whereas there was no significant correlation between the PVD score and the rating score for mask-free Black faces (*r* = 0.317, *p* = 0.081). For the White and non-Japanese Asian faces, there were no significant correlations between the PVD score and the rating scores of masked and mask-free faces (masked White: *r* = 0.091, *p* = 0.623; masked non-Japanese Asian: *r* = 0.075, *p* = 0.696; mask-free: White: *r* = 0.043, *p* = 0.815; mask-free non-Japanese Asian: *r* = −0.064, *p* = 0.739). For the perceived infectability and germ aversion subscale scores, we also computed Pearson’s correlation coefficients. A positive correlation between the germ aversion score and the rating score for masked Black faces was significant (*r* = 0.369, *p* = 0.040). However, there was no significant correlation between the germ aversion score and the rating score for mask-free Black faces (*r* = 0.298, *p* = 0.102). Furthermore, there were no significant correlations between the germ aversion score and rating scores for masked and mask-free White and non-Japanese faces (masked White: *r* = −0.046, *p* = 0.805; mask-free White: *r* = −0.043, *p* = 0.814; masked non- Japanese Asian: *r* = −0.184, *p* = 0.338; mask-free non-Japanese Asian: *r* = −0.157, *p* = 0.414). No significant correlations were found between the score for perceived infectability and the attractiveness scores for other-races mask-free and masked faces (mask-free Black: *r* = 0.163, *p* = 0.380; masked Black: *r* = 0.160, *p* = 0.388; mask-free White: *r* = 0.111, *p* = 0.549; masked White: *r* = 0.185, *p* = 0.316; mask-free non-Japanese Asian: *r* = 0.058, *p* = 0.763; masked non-Japanese Asian: *r* = 0.228, *p* = 0.233). We show the graphs of the correlation results between the rating scores and PVD scores in Supplementary material C.

In general, there were no significant negative correlations between the PVD, germ aversion, and perceived infectability scores and the rating scores of mask-free other-races faces. These results were inconsistent with our prediction that people who report higher PVD scores should perceive mask-free other-races faces as less attractive relative to others who report lower PVD scores. Although the rating score for Black masked faces was positively correlated with the PVD and germ aversion scores, our replication study using a crowdsourcing service with the identical procedure as the original study (*N* = 46, 22 females, *M* = 40.67 years, *SD* = 9.96) did not identify significant positive correlations [PVD score: ρ (46) = −0.035, *p* = 0.815; germ aversion score: ρ (46) = −0.038, *p* = 0.801]. Therefore, care should be taken when interpreting these positive correlations.

## 4. Discussion

In the present study, we investigated the effects of two different factors on the attractiveness rating of masked faces. The first factor involved interpolation of the masked area. We predicted that the facial area occluded by a sanitary mask would be interpolated differently based on moderate-attractiveness templates between own-race and other-races faces. We argue that for own-race faces, respondents would have shaped a moderate-attractiveness template of good quality through experience and apply this template to interpolate the occluded area of the masked faces. Note that this moderate-attractiveness template differs from an averaged representation (Rhodes et al., 1987; Rhodes and Tremewan, 1996; Rhodes et al., 2007) of own-race faces that reflects developmental stability and heterozygosity, thus resulting in improvements in attractiveness when integrated into a whole-face representation. However, the moderate-attractiveness template represents a facial knowledge that is neither extremely attractive nor extremely unattractive but instead represents an average level of attractiveness. This results in an interaction with baseline attractiveness when the occluded area is interpolated using a moderate-attractiveness template. Specifically, interpolating masked attractive faces based on a moderate-attractiveness template should reduce attractiveness, whereas interpolating those of unattractive faces should increase attractiveness. This moderate-attractiveness interpolation process should not apply to other-races faces because we do not have counterpart templates for other-races faces due to lack of experience accumulated knowledge. Instead, the template used for interpolating other-races faces is biased toward highly attractive faces because of sparse exposure to other -races faces. Therefore, interpolation using this template should result in an improvement in attractiveness regardless of baseline attractiveness.

The second factor is an exclusive attitude toward the other-races. Because an exclusive attitude toward an other-races population is positively related to concern regarding the COVID-19 threat (e.g., Yamagata et al., 2020; Adam-Troian and Bagci, 2021; Mula et al., 2022), mask-free other-races faces should be rated less attractive if this factor is in effect. In particular, we predicted a negative correlation in that people who report higher PVD scores should perceive mask-free other-races faces as less attractive relative to others who report lower PVD scores, because of the association between infection-avoidance tendencies and strong exclusionary attitudes toward persons from abroad (Yamagata et al., 2020).

For the first interpolation-related factor, the present results supported the explanation of interpolation based on a moderate-attractiveness template and did not support that based on interpolation by an average representation. Specifically, mask-wearing did not simply improve perceived attractiveness. Rather, we found an interaction effect. Masked attractive own-race faces were rated less attractive than mask-free faces, whereas masked unattractive own-race faces tended to be rated more attractive than mask-free faces. By contrast, masked other-races faces were rated as more attractive than mask-free other-races faces regardless of the level of facial attractiveness. Importantly, the premise of the moderate interpolation hypothesis that the Japanese participants have opportunities to form a fine-detailed template for own-race faces, although they do not have such chance to train templates for other-races due to infrequent encounter to other-races faces was supported. Regarding the second factor of exclusive attitudes toward other-races, there were no negative correlations between the PVD, germ aversion, and perceived infectability scores and the rating scores for mask-free other-races faces. Overall, the race the faces belonged to was a primary factor that affected the attractiveness rating and the effect from the presence or absence of an exclusive attitude was negligible.

Although it is possible that differences between the two datasets influenced our results, we consider this unlikely because we conducted between-subjects experiments for each race and the participants could not compare images between the datasets. Moreover, the participants used different laptop computers for the web-based experiments and it was not feasible to control/match image quality for each race. Furthermore, there was no significant correlation between display size and attractiveness ratings in Experiments 3 and 4, which were conducted on the participants’ personal computers (Supplementary material D). Our conclusions are based on the results obtained for images of varying quality that were available during the COVID-19 pandemic (when the study was conducted). The differences in image quality and presentation size among image sets were limitations of the present study, although it is unlikely that these differences alone could explain the findings. Ideally, image sets comprising photographs obtained under similar lighting conditions, and with similar resolutions, should have been used for rigorous testing of the effects of wearing masks for the different races.

The present findings supporting that the interpolation process is based on a moderate-attractiveness template can be bolstered by the following two theories. First, interpolation based on moderate attractiveness could explain the results of the present study better than the hypothesis relying on interpolation by average representation and regression to the mean. The moderate interpolation hypothesis posits that viewers shape their moderate-attractiveness template based on the distribution of their own experience and interpolate the occluded area based on this template. Specifically, when an occluded face belongs to the own-race, the distribution of the viewer’s experience is similar to the real-world facial attractiveness distribution because the viewer experiences a variety of own-race faces. Therefore, unattractive occluded own-race faces are perceived as more attractive than the original faces, whereas attractive occluded own-race faces are perceived as less attractive than the original faces. When the occluded face is from an other-races, the distribution of the viewer’s experience is biased toward highly attractive faces and is different from the real-world facial attractiveness distribution because their exposure is limited to attractive faces. Therefore, occluded faces are perceived as more attractive than the original faces regardless of baseline attractiveness. As mentioned above, moderate-attractiveness interpolation explains the results of the present study.

On the other hand, according to the average interpolation hypothesis stating that viewers interpolate the occluded area based on an average representation perceived as attractive, attractive occluded faces should be perceived as more attractive than the original faces, but this hypothesis is inconsistent with the present result for occluded attractive own-race faces. Moreover, with regression to the mean, occluded facial attractiveness is standardized by a decreasing cue for judging facial attractiveness, thus attractive occluded faces should be perceived as unattractive relative to the original faces, but this hypothesis is also inconsistent with the present results for occluded attractive other-races faces.

As a second theory, the moderate interpolation hypothesis may reduce the error between the perception of occluded faces and original faces. Living organisms, including humans, should behave in a way that minimizes the error between their own predictions and the external state as perceived through their sensory systems in their daily lives (e.g., Friston, 2010; Ohira, 2017). Therefore, the interpolation of a moderate-attractiveness template is reasonable because the template represents the statistically most probable samples and was advantageous in reducing modification errors between predictions and real faces when the faces were revealed.

Experience with facial categories (own- or other-race) could influence the development of a real-world moderate attractiveness template. It has been suggested that template formation depends on the viewer’s experience. Apicella et al. (2007) reported that the Hadza people from Northern Tanzania did not prefer an average European face, but did prefer an average Hadza face. This preference for an average face of their own race was attributed to the fact that the Hadza were not sufficiently exposed to European faces to form an accurate impression regarding what constitutes an average European face. Furthermore, 5-year-old children were less influenced by average facial attractiveness than adults (Vingilis-Jaremko and Maurer, 2013). These findings suggest that there is no universal template for attractiveness; frequent encounters with different races are required to generate the average face template. In other words, increased encounters with a specific group of faces would generate knowledge. Encounters with particular types of other-race faces, such as those of movie stars and athletes, may lead the viewer to believe that those types of faces are common within the race in question. This results in a moderate-attractiveness template for other races biased toward highly attractive faces.

One limitation of the present study was the potential variation in facial attractiveness among image datasets. In particular, the highest attractiveness score for mask-free faces was 60 for own-race faces, with lower scores (around 30–50) seen for the other races. Previous studies have reported a preference for own-race faces over those of other races (Fisman et al., 2008; Liu et al., 2015; Rodway et al., 2019). Furthermore, own-race individuals are encountered more frequently than those of other races, which leads to the “Cross-Race effect,” i.e., own-race faces can be discriminated more precisely than other-race faces. Therefore, attractiveness ratings for own-race faces are likely to vary more widely than those for other-race faces.

In conclusion, the present study examined the moderate interpolation hypothesis, and the results were consistent with the hypothesis. The hypothesis reconciles the mixed results of ratings of occluded facial attractiveness in previous studies. It should be noted that the present study only focused on Japanese participants. Therefore, future research should examine the moderate interpolation hypothesis for other races. For example, in a previous study, Cardiff University students perceived occluded faces, varied as to race, as being more attractive than unoccluded faces (Hies and Lewis, 2022). In another study, Dudarev et al. (2022) examined attractiveness ratings for masked White and Asian faces; they found that participants’ attitudes toward mask-wearing were strongly associated with political orientation, thus affecting the judgment of attractiveness regarding masked faces. Given the variation in the progression of COVID-19 spread, the reactions to mask-wearing people differ considerably among countries. To avoid this contextual influence, it is important to occlude the face with a neutral object that does not evoke association with any attitudes (e.g., political attitude). Pursuing these research questions will contribute to a better understanding of the mechanism of why we observe occluded faces as more or less attractive.

## Data availability statement

The datasets presented in this study can be found at: https://osf.io/5yzmh/.

## Ethics statement

The studies involving human participants were reviewed and approved by the Ethical Review Board of Hokkaido University (31–2; 3–02). The participants provided their written informed consent to participate in this study.

## Author contributions

MK and YM processed the photographs and created stimuli. MK programmed the study and collected and analyzed the data. All authors designed the study and contributed to the manuscript text.

## Funding

JK was funded by Grants-in-Aid for Scientific Research from the Japan Society for the Promotion of Science (No. 20H04568). This work was partially supported by Graduate Grant Program of Graduate School of Humanities and Human Sciences, Hokkaido University, Japan and JST SPRING, Grant Number JPMJSP2119 to MK.

## Conflict of interest

The authors declare that the research was conducted in the absence of any commercial or financial relationships that could be construed as a potential conflict of interest.

## Publisher’s note

All claims expressed in this article are solely those of the authors and do not necessarily represent those of their affiliated organizations, or those of the publisher, the editors and the reviewers. Any product that may be evaluated in this article, or claim that may be made by its manufacturer, is not guaranteed or endorsed by the publisher.

## Footnotes

^1^This can be analogously described in terms of height. We have a relatively accurate conception of the height distribution among people of our own race, but our perception of the height distribution among people of other races is likely to be influenced by images of actors or athletes, leading us to imagine higher average heights.

^2^ http://www.chicagofaces.org/
