# Supplementary material for: Occlusion of faces by sanitary masks improves facial attractiveness of other races
Source: Front Psychol. 2023 Jan 12;13:953389. doi: 10.3389/fpsyg.2022.953389 (PMC9878602; doi:10.3389/fpsyg.2022.953389)
Supplement: Supplementary file 1 [file Table_1.docx]

Supplementary Material

**A: Correlations between rating scores and PVD scores of unpublished data.**

We previously examined the correlation between rating score and PVD scores in data completely unrelated to the present study.

**Method**

**Participant**

Fifty-nine Japanese undergraduate and graduate students (*M* = 19.98 years, *SD* = 1.61, 30 females) participated. The study was conducted from May 19, 2020 to July 30, 2020.

**Apparatus and Stimuli**

The apparatus and stimuli were identical to those of Experiment 1 except for the number of stimuli, presentation size of stimuli and mask color. A total of 66 images were selected from a homemade database of young female Japanese facial images (Kawahara & Kitazaki, 2013). Twenty-two images were included in three attractiveness categories (low, middle, and high). The images were presented with a height of 6.85 cm and width of 9.10 cm on a 14-in. laptop screen. Mask color was white and black. Mask color was a between-subjects factor.

**Procedure**

Procedure was same to those of Experiments 2-4.

**Statistical analysis**

Analysis was conducted using R software (version 4.2.1). Three of the participants were excluded because they did not answer the PVD questionnaire. We computed the sum of scores for each participant.

**Results**

Cronbach’s alpha for all 15 items was .84, Cronbach’s alpha for the perceived infectability subscale was .91, and Cronbach’s alpha for the germ aversion subscale was .76. We calculated Pearson’s correlation coefficients between the PVD score and rating scores of masked and mask-free faces. There were no significant correlations between the PVD score and the rating score for masked and mask-free faces (masked: *r* = −.208, *p* = .123; mask-free: *r* = −.084, *p* = .538). For the germ aversion and perceived infectability subscale scores, we also computed Pearson’s correlation coefficients. There were no significant correlations between the germ aversion score and the rating score for masked and mask-free faces (masked: *r* = −.079, *p* = .558; mask-free: *r* = −.021, *p* = .872). Furthermore, there were no significant correlations between the perceived infectability score and the rating score for masked and mask-free faces (masked: *r* = −.239, *p* = .075; mask-free: *r* = −.105, *p* = .436).

**B: Attractiveness ratings after rearranging the stimuli for people living in Japan**

We rearranged the faces chosen from the Chicago Face Database into three attractiveness categories for 153 adults (*M* = 39.58 years, *SD* = 10.35; 76 females) living in Japan, recruited from the crowdsourcing service CrowdWorks. In total, 35.4% (51/144) photographs were reclassified into attractiveness categories different from those based on the Chicago Face Database. Supplementary Table 1 shows the details of this reclassification process for Experiments 2–4. The mean rating scores were plotted as a function of baseline attractiveness for the masked (solid line) and mask-free (dashed line) faces in each panel (Supplementary Figure 1). A two-way (baseline attractiveness × mask presence) within-subjects analysis of variance of the rating for Black, White, and non-Japanese Asian faces revealed significant main effects of baseline attractiveness (*F*s (2, 62) > 55.532, *p*s < .001, η_p_^2^s > .641) and mask presence (*F*s (1, 31) > 19.949, *p*s < .001, η_p_^2^ > .391). The Holm’s tests revealed that attractiveness rating increased with an increase in baseline attractiveness (*t*s (31) > 5.580, *p*s <.001, *r*s > .708). The interaction between baseline attractiveness and mask presence was not significant (*F*s (2,62) < 2.753, *p*s > .071, η_p_^2^s < .081).

Supplementary Table 1

The detail of re-arranged stimuli

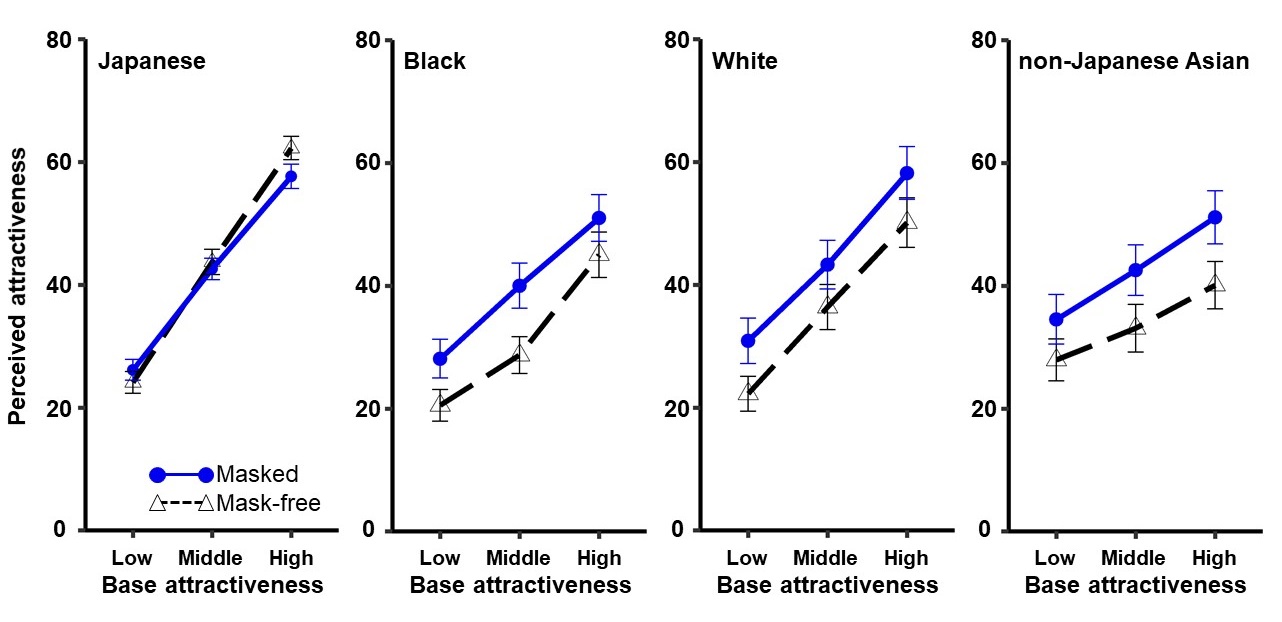
Supplementary Figure 1. Attractiveness ratings for masked and mask-free faces in each experiment. From left to right: Japanese, Black, White, and non-Japanese Asian faces. The graph for Japanese individuals is provided for comparison with those for other races with reclassified stimuli. Stimulus reclassification was not performed for the Japanese faces. Error bars represent standard error.

To summarize, although the stimuli were rearranged into attractiveness categories based on the Chicago Face Database attractiveness ratings for adults living in Japan, facial attractiveness ratings for the other races increased when a sanitary mask was worn, regardless of the facial attractiveness level. Importantly, the results were virtually identical to the original ones.

**C: Graphs of the correlations between the rating scores and PVD scores**


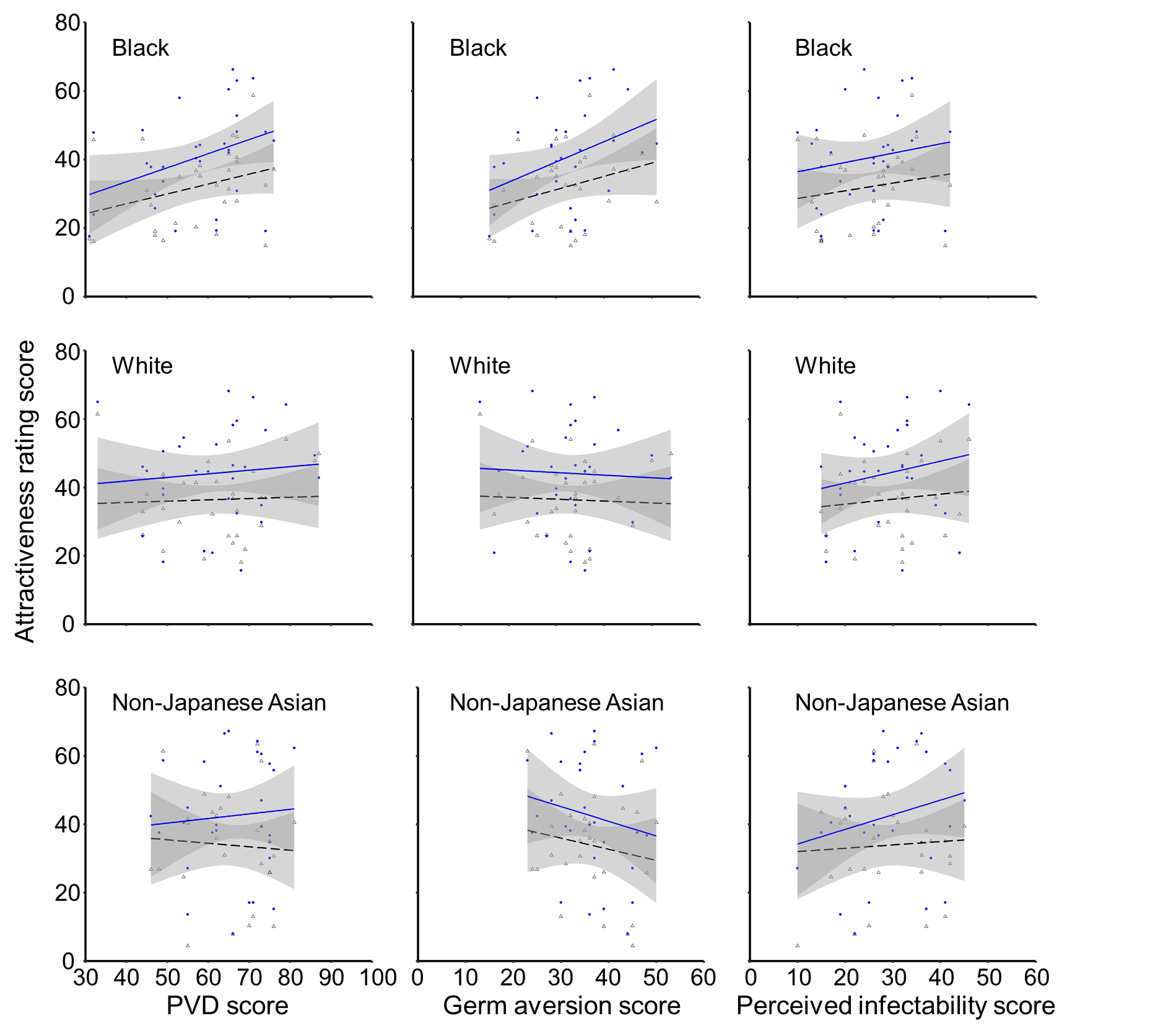

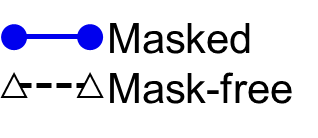


Supplementary Figure 2. Correlation plots between the PVD score and attractiveness rating scores of masked and mask-free faces with respect to race. The left column shows the correlations between the PVD score and attractiveness rating scores of masked (blue solid lines and blue circles) and mask-free (black dashed lines and black triangles) individuals. The middle column shows the correlations between the germ aversion scores and attractiveness rating scores of masked and mask-free individuals. The right column shows the correlations between the perceived infectability scores and attractiveness rating scores. The gray band represent the 95% confidence interval.

**D: Correlations between ratings and display size on the participants’ personal computers**

In Experiments 1, 3, and 4, stimuli were presented to the participants via a web browser running on their personal computers. Therefore, the size of the presented stimuli varied among the participants. We performed a post-hoc correlation analysis to confirm whether display size affected the attractiveness ratings. The analyses were performed in Experiments 3 and 4, in which there was no significant interaction between baseline attractiveness and mask presence.

There was no significant correlation between display size and attractiveness ratings for masked or mask-free faces (mask-free non-Japanese Asians: *r* = −.042, *p* = .818; mask-free Whites: *r* = .006, *p* = .970; masked non-Japanese Asians: *r* = −.173, *p* = .343; masked Whites: *r* = −.170, *p* = .351). Moreover, there was no significant correlation between display size and differences in attractiveness according to mask presence (non-Japanese Asians: *r* = −.192, *p* = .290; Whites: *r* = −.251, *p* = .164).

The post-hoc analysis revealed no negative or positive correlation between display size and attractiveness ratings, suggesting that the effect of display size was negligible.
